# Training Schools and Private Nursing

**Published:** 1909-05-29

**Authors:** 


### NURSING ADMINISTRATION.

### TRAINING SCHOOLS AND PRIVATE NURSING.

It is remarkable that although nine-tenths of the nurses trained in hospitals become, for some part of their career at any rate, private nurses, no part of the curriculum of the training-school is directed to fitting them for the special duties and difficulties of private nursing. The reason for this is not far to seek. The training of a nurse is conducted exclusively by sisters who have themselves had no experience whatever in this branch of work. It is supervised by a matron who has in all probability never taken a private case, and the problems which will engage the nurse on leaving hospital are a sealed book to her superiors as well as to herself. Do not the hospitals owe a duty to the public in this connection? The service rendered by hospitals in fitting women to undertake the nursing of patients in private life is often brought forward as a reason for generous support, and it is just that this should be so. Every institution to which a good training school for nurses is attached can point to large expenditure incurred, quite beyond the immediate needs of the wards, in order to make the training of nurses entirely efficient.

In many establishments the number of probationers is maintained at a figure greatly in excess of requirements, expressly that as many women as possible may be afforded the necessary training. But having gone so far, is it not incumbent on hospital authorities to go a little farther? At present the trained nurse leaves the wards confessedly very ill-prepared for the widely different duties which await her in the sick-room, and unless she have exceptionally quick wits and power of adaptability her deficiencies are instantly exposed. Even in the actual services to be performed for the patient, constituting what may be called nursing proper, she is likely to go astray, and nearly all the complaints which are freely levelled against private nurses may be referred back to a hospital attitude towards the patient, entirely out of place in private practice. The patients in hospital are not so much individuals as part of a large scheme. With the utmost consideration and the tenderest humanity among those who care for them, it is evident that the patients' idiosyncrasies can be allowed little play. Things which have to be done for them are done when it is convenient for the common good of the ward, and are necessarily performed at lightning speed—the efficiency of a hospital nurse being measured by her rapidity of action. All this is quite out of place in a private room where the attentions of the nurse are concentrated on one patient, and where an atmosphere of haste is highly offensive. In hospital the nurse is the patient's superior; she assumes command, and either controls with a high hand, or talks down to those on whom she waits. She acquires either an impassive manner born of concealed hurry, or what is known as the "dearie" manner, and both are about equally distasteful to many people, who see no reason why the fact of being ill should reduce them to be treated like knaves or fools. Again, it is quite true that probationers during their time of training are entirely excluded from the outside world, and this, as Dr. Jane Walker recently observed, has a bad effect upon them as private nurses. It frequently happens that prior to entering hospital they have had no experience of other people's houses, and it is not surprising that, plunged without preparation into unfamiliar surroundings, varying from a public-house to a ducal mansion, they should be guilty of blunders which serve as material against nurses in general. Lastly, the relations between doctor and nurse in private practice and in the hospital ward differ often very widely. Nurses are bound to the very strictest observance of an etiquette which has never been properly defined to them, and offend usually because the circumstances in which they find themselves are outside their experience. In all these aspects of private work the nurse sins more often in ignorance than in intention. There is little doubt that it is the defective preparation received in her training school which is answerable for much which renders her unpopular in the outside world.

It is time that the heads of training schools began to recognise distinctions in the work for which they are training probationers. If during the first two years the training can run on even lines, the basis of a nurse's training being the same whatever line she may subsequently take up, it is beginning to be perceived that during the last year or two years she should be encouraged to choose her future career and begin seriously to qualify for it. The private nurse will not need to make herself proficient in all the subjects necessary to a future matron. But she has special trials ahead of her, and it is not creditable to the training school which certificates her if she is found unprepared to meet them. Very few hospital sisters, as we have said, are qualified to instruct nurses as to their duties in private sick-rooms, or to give them a rule of conduct in other people's houses small and large. But it should not be difficult to procure one member of the staff experienced in the ways of the world, and in the problems of private nursing, who could lecture to those among the third or fourth year probationers who had expressed their intention of becoming private nurses, and the very fact of a regular course of study directed to this end would introduce a new spirit among the nurses thus instructed. There is no doubt that paying wards would greatly help out such a course, though they cannot obviate the necessity for it. There is a whole code of conduct necessary to be mastered by the woman who intends to make a success in private nursing, and the hospital which will initiate a course of this nature will confer a benefit not merely on its own probationers and their future patients, but on the nursing world, which suffers deeply in reputation from the errors of uninstructed women.

